# Facial and genital color ornamentation, testosterone, and reproductive output in high-ranking male rhesus macaques

**DOI:** 10.1038/s41598-024-52400-0

**Published:** 2024-01-31

**Authors:** Gisela Sobral, Constance Dubuc, Sandra Winters, Angelina Ruiz‑Lambides, Melissa Emery Thompson, Dario Maestripieri, Krista M. Milich

**Affiliations:** 1 Department of Anthropology, Washington University in St. Louis, St. Louis, MO USA; 2 Instituto de Biodiversidade e Sustentabilidade (NUPEM/UFRJ), Macaé, RJ Brazil; 3 Department of Anthropology, New York University, New York, NY USA; 4 Organismal and Evolutionary Biology Research Programme, Faculty of Biological and Environmental Sciences, University of Helsinki, Helsinki, Finland; 5 Caribbean Primate Research Center, University of Puerto Rico, San Juan, Puerto Rico; 6 Department of Anthropology, University of New Mexico, Albuquerque, USA; 7 Institute for Mind and Biology, University of Chicago, Chicago, IL USA

**Keywords:** Sexual selection, Animal behaviour, Animal physiology

## Abstract

Males in many vertebrate species have colorful ornaments that evolved by sexual selection. The role of androgens in the genesis and maintenance of these signals is unclear. We studied 21 adult high-ranking male rhesus macaques from nine social groups in the free-ranging population on Cayo Santiago, Puerto Rico, and analyzed facial and genital skin luminance and redness, fecal androgens, rates of mating behaviors, and offspring sired. Facial and genital coloration varied in relation to age, mating behavior, reproductive success, and testosterone concentration. Our results indicate that skin coloration in high-ranking male rhesus macaques is a sexually-selected trait mediated by androgens. These results add to the growing literature on the proximate and ultimate causes of male sexual signals and highlight the need to examine how these characteristics change with age in other species.

## Introduction

Sexually-selected signals are widespread in animals and come in many forms, including armaments, colorful ornaments, songs, pheromones, and more^1,2^. Honest signals are generally costly, such that only high-quality individuals can afford elaborate ornaments^3,4^. Low-quality individuals are more likely to produce cheaper, less elaborate, or sometimes dishonest signals^3,5, 6^. This pattern has been shown by a large body of research demonstrating links between signal quality and genetic quality or condition, especially in males^7–10^. Females who choose to mate with well-ornamented males can benefit directly from their courtship behavior (e.g., if it involves providing food, space, protection, or other services) and may be more likely to produce offspring of high genetic quality^3,7, 11, 12^.

Although many male mammals are equipped with armaments such as antlers, fangs, and claws^11,13–17^, relatively few aside from some primate species present colorful ornamentation^18^. Perhaps the best example of mammalian color ornamentation is the male mandrill, which has bright red and blue facial patterns^19^. Males in other primate species also have bright coloration in their faces (e.g., uakaris^20^; rhesus macaques^18^), chests (e.g., geladas^21,22^), or genitals (e.g., vervet monkeys^23,24^). In some species, coloration reflects male age and rank^25^ and brightness reflects health^20^ and blood flow^26^.

Many primate species live in groups with dominance hierarchies that result in uneven access to resources, with the result that higher-ranking males are generally in better body condition than lower-ranking ones^27^. However, there is no clear relationship between male rank, body condition, and ornamentation. Coloration has been correlated with social status in vervet monkeys^28^ and mandrills^19^, but other studies found opposing or no relationship between social status and coloration^29,30^. Given that sexually-dimorphic coloration in primates evolved independently multiple times, it may function and be regulated differently in different species^31^, and factors other than rank can impact coloration^29,32^. Additionally, some aspects of coloration in male primates are sexual signals used in female choice^33,34^, while others are associated with signalling to other males as part of intrasexual competition^34,35^.

Testosterone and other androgens are key hormones in regulating the expression of many forms of male ornamentation and other sexually-dimorphic traits^18,25,36–39^. High testosterone production, however, can suppress immune function^6^ such that in many species, only high-quality individuals are capable of maintaining their health while producing costly, testosterone-dependent ornaments^6^. High-ranking males generally maintain higher concentrations of testosterone^40–42^; therefore, coloration is often correlated with rank position. However, there are exceptions to this pattern^43^.

The rhesus macaque (*Macaca mulatta*) is one of the primate species in which male individuals present facial and genital ornamentation^30,44^. This species also has clear linear dominance hierarchies related to age classes for males^45^. However, these hierarchies can be unstable at times and do not always follow the order of succession^46,47^. High-ranking males spend more time in consortship with females^48^ and have higher rates of copulation and ejaculation^49^, resulting in greater reproductive success than low-ranking males^48,50,51^. In rhesus macaques, higher-ranking males do not necessarily have redder faces or genitals^30,33,52^. Males with dark red faces, however, receive as much attention from females^33^ (as well as from other adult males^53^) as high-ranking males do^54^.

Although one would expect high rank and red coloration to be both associated with high testosterone, this is not necessarily the case. Many studies of rank and testosterone in male rhesus macaques have produced conflicting results^55–59^, and the only study to date of rank and color in this species has produced negative results^30^, despite the fact that red color is hypothesized to be androgen-dependent^18,36,39^. Age could potentially be a variable complicating the relationship between rank, reproduction, hormones, and coloration, as age is known to be associated with each of these factors^60,61^.

Previous research has found that males who are both high-ranking and dark in color have higher fecundity than other males^62^. Thus, in this study, we focused on top-ranking males, which allowed us to investigate further whether variation in skin color among these males explains variation in their mating behavior and reproductive success. By focusing on males who are in similar social and sexual contexts, we decreased the sources of variation that can otherwise impair our ability to understand the causes and consequences of skin color variation. The aim of this paper is three-fold: first, to understand how androgens and color ornamentation (measured as facial and scrotal redness and luminance) correlate; second, to test if androgens and color vary by rank position and age among top-ranking male rhesus macaques; and third, to examine if reproductive variation (namely rates of consortship, mounting, and ejaculation and the number of offspring produced by each male), was predicted by color and testosterone concentrations, independent of age or rank. Based on previous research, we did not expect a relationship between rank and color^30,33,54^ or between rank and testosterone^56,60^. However, we did expect that facial and genital skin color would vary with androgen concentrations and male reproductive effort. We expected that males with higher testosterone and dark red faces would have higher rates of consortship, mounting, and ejaculation and sire more offspring. We also expected that older males would have lower testosterone and reproductive success^60,61^.

## Material and methods

### Study animals

The study was conducted on the Cayo Santiago rhesus macaques, a semi free-ranging population located on an island in Puerto Rico. The population is provisioned with food and water and has been monitored since its foundation in 1938^63^. At the time of this study, this population consisted of approximately 1200 individuals distributed in multimale-multifemale groups of various sizes^47^. Birth records, genetic data, and pedigrees of the island individuals are available, allowing us to determine reproductive success of males^63^. Data for this study were collected during the mating season of 2013 (February to July). We collected behavioral data, fecal samples, and images from 21 males (between 7 and 21 years old) belonging to nine groups. These males were selected based on their high rank (being one of the top three highest-ranking males in a given group) at the onset of the study^47^. Rank number varied from 1 (alpha) to 2 (beta) or 3 (gamma) for males in established groups and rank 4 was used for three males that were alpha (n = 2) and beta (n = 1) males in two small, recently formed groups that were not well-established. The study was approved by the IACUC of the University of Puerto Rico, Medical Sciences Campus (Protocol No. A0100108). All methods were performed following the relevant regulations and guidelines.

### Behavioral data collection

Detailed information on behavioral data collection can be found in Milich et al.^61^. Briefly, animals were followed for five days a week from March to July 2013, and we conducted 10-min focal follows on each male. If males engaged in consortship, focal follows would last up to 60 min. During focal follows, we collected scans every two minutes. Consortship was defined as an extended association between a male and a female with both doing activities together (travelling, affiliative behaviors, mountings etc.). During these consorthips, we noted all occurrences of sexual behavior, such as mounting and ejaculation, *ad libitum*^61^.

### Hormonal assay

Sample collection and extraction followed Milich et al.^64^. Briefly, we collected fecal samples opportunistically during focal follows and stored them on ice while in the field and then at − 20 °C until extraction. Samples were collected into tubes immediately after defecation by an identified focal individual. Using 0.5 g (± 0.03 g) of fresh samples, we employed the extraction protocol of Palme^65^ using 80% methanol. We measured immunoreactive testosterone in the resulting 234 fecal extracts ($\overline{X}$= 11 samples/male) using enzyme immunoassays (EIAs) at the University of New Mexico, with reagents and protocols provided by the Clinical Endocrinology Laboratory at the University of California at Davis (Antibody R156/7). This protocol is the most widely used assay for the determination of testosterone in mammalian feces, having been validated across a wide range of taxa, including in primates (*Mandrillus sphinx*^39^; *Colobus vellerosus*^66^; *Gorilla beringei beringei*^67^). An alternative assay for epiandrosterone has been preferred for macaques based on an in vivo experiment that found negligible concentrations of excreted testosterone in the feces of a single long-tailed macaque^68^. However, we found the testosterone assay produced highly correlated results to the epiandrosterone assay (r = 0.55, N = 50, *p* < 0.0001), but with increased reliability. Interassay CVs were 12.7% for a low sample pool and 9.6% for a high sample pool and the intra-assay CV of duplicates was 5.7%.

### Color data collection

Facial and genital photographs were taken in March and April 2013. From the photos, we measured red intensity and luminance non-invasively. These measures are similar to measures in humans and domesticated animals that are categorized by brightness and redness (e.g.^69^). Photos were compared to a color standard (X-rite ColorChecker passport) to account for ambient light color and intensity.

Each photograph was taken 1–3m away from the male while he was sitting or standing still in a clear location and not in contact with another individual with a Canon EOS Rebel T2i 18-megapixel camera with a CMOS APS sensor and an EF-S 55–250 mm f/4–5.6 IS lens^70–72^. Using the sequential method^70–72^, a second and a third photo of the color standard (which included a neutral white patch) held by an assistant in the same position as the skin area had been photographed immediately afterwards using the same camera settings. Whenever possible, we took multiple pictures of the same male.

Skin coloration was quantified using methods previously described elsewhere^33^. Briefly, images were converted to 16-bit TIFF files using DCRAW^73^, and average red (R), green (G), and blue (B) measurements were taken from a fixed portion of the face and the neutral white patch from the color standard. RGB values were computationally transformed from the camera’s color space to rhesus color space using standard visual modelling methods^74,75^, resulting in estimates of rhesus long (LW, 565 nm), medium (MW, 535 nm), and short (SW, 431 nm) wavelength photoreceptor catches (data from^76,77^. Two measures of facial coloration were then calculated: the red-green opponency (R-G) channel (LW − MW)/(LW + MW), and the luminance channel (LW + MW)/2^78^. Both R-G and luminance were calculated for every image, averaged within a series of images at a given time point, and averaged across series within a day when applicable. Given that color measures could change slightly from day-to-day (redness SD < 0.03 and luminance SD < 0.1) we averaged color values monthly. These methods follow previously validated protocols for use in primates^70^ that have previously been shown to produce reliable color measurements within this population^33^.

### Number of offspring sired

As previously described in Milich et al.^61^, the number of offspring produced in 2013 were calculated from the long-term database managed by the Caribbean Primate Research Center. Briefly, samples for genetic analysis to determine offspring paternity were collected when the offspring were yearlings, meaning that infants who died prior to this sampling period were not included in the calculations. In 2013, infant mortality was 13% of all infants born, and we cannot account for who sired those offspring.

### Statistical analysis

For the mating behavior measures, we used three measures and calculated the percentage of days in which a male was observed (1) consorting, (2) mounting, or (3) ejaculating with a female partner (hereafter ‘rates’). Reproductive success was determined by the number of offspring produced by a given male in 2013. The age of each male was calculated from the long-term birth records for Cayo Santiago. Variables were tested for normality using Shapiro–Wilk tests and variables were normally-distributed, except for age, rank, and the offspring produced.

First, we checked the correlations between ornamentation and testosterone with Pearson’s correlation. Given that color measures were highly correlated, we used one measure of coloration at a time and one reproductive measure as the dependent variable at a time. For our second aim, to understand whether and how androgens and color ornamentation vary with rank and age among top-ranking male rhesus macaques, we tested if face redness and luminance and scrotal redness and luminance were predicted by testosterone, rank or age with linear models (facial/scrotal coloration ~ testosterone + rank + age). We also compared model fit including and excluding male ID as a random factor with ANOVA test. Because male ID did not affect the model, we opted for the most parsimonious approach^79^ of using the simplest model (i.e., those without male ID as the random factor).

Third, to examine whether skin color, testosterone concentrations, rank, and age predicted reproductive measures (namely rates of consortship, mounting, and ejaculation and the number of offspring produced by each male), we employed several models to investigate these relationships. We used one reproductive measure at a time: reproductive variation measure ~ facial/scrotal coloration + testosterone + rank + age.

In all models, we used monthly average values for skin color, testosterone concentrations, and rates of consortship, mounting, and ejaculation for each male. Because rank, age, and offspring sired did not change monthly, we used a single measure. We used *lme4* package with Gaussian family distribution for most models, except the number of offspring sired, where we used the Poisson distribution given that the residual deviance divided by the degrees of freedom was close to 1 (1.14). We provide Akaike Information Criteria (AIC), as well model fit R^2^ for each model. We ran all analyses in R v4.0.3.

## Results

### Variation between facial coloration, genital coloration, and androgen concentrations

There were significant negative correlations between luminance and redness for both facial (r =  − 0.46; *p* < 0.001) and genital skin (r =  − 0.46; *p* < 0.001), meaning that redder skin was also darker. There was a strong positive correlation between facial and genital redness (r = 0.52; *p* < 0.001) and a weaker correlation between facial and genital luminance (r = 0.3; *p* = 0.03), meaning that skin color and luminance in both areas co-varied.

Testosterone was significantly positively correlated with face luminance (r = 0.36; *p* = 0.009) and negatively correlated with face redness (r =  − 0.27; *p* = 0.05), meaning that males with higher testosterone concentrations were paler and less red than other males. Similarly, testosterone was also positively correlated with scrotal luminance (r = 0.44; *p* = 0.006), but not with scrotal redness (r =  − 0.2; *p* = 0.2).

### Effect of rank position and age on androgens and color ornamentation

**Figure 1:**
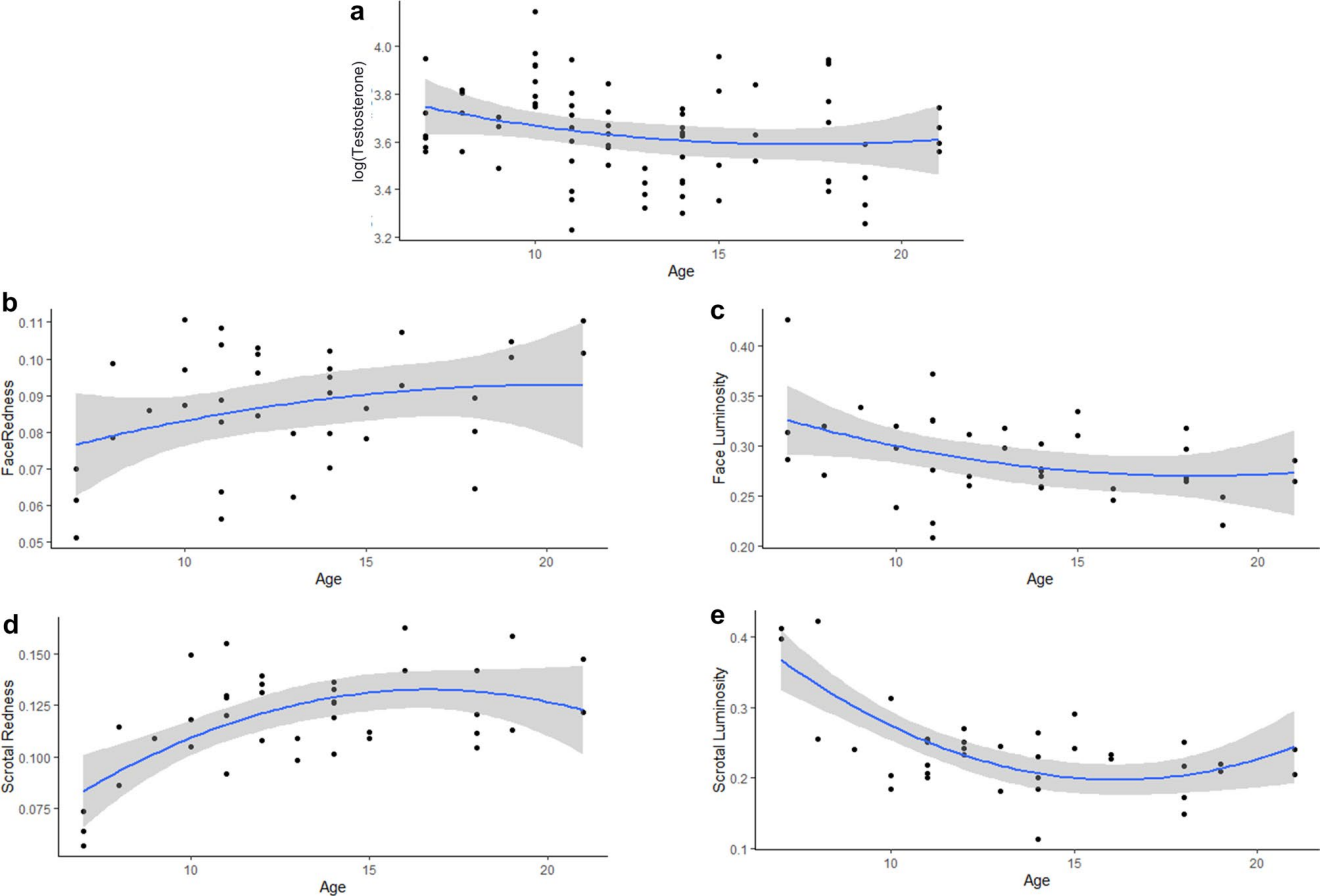
Relationships between age and testosterone (**a**), face redness (**b**), face luminance (**c**), scrotal redness (**d**), and scrotal luminance (**e**). Effects obtained with generalized linear models. Trendlines are polynomial of order 2.

Among high-ranking males, rank was not a significant predictor of facial or scrotal coloration or testosterone (Table 1). These findings are potentially biased because all males were high ranking. However, age was an important predictor of several measures (Fig. 1; Table 1). Face redness varied by age (Fig. 1b; Table 1), and face luminance was negatively associated with age, with older males having lower luminance (Fig. 1c; Table 1). Scrotal coloration was predicted by age, with older males having redder and darker scrotum (Fig. 1d,e; Table 1).

**Table 1:** Models using rank and age to predict face redness, face luminance, scrotal redness scrotal luminance, and testosterone in male rhesus macaques, *Macaca mulatta*, living on Cayo Santiago, Puerto Rico.

Model	*p* Value model	AIC	R^2^	Significant effect
Face Redness ~ Age + Rank	0.1331	− 319.879	0.1827	Age (+)
Face Luminance ~ Age + Rank	0.1788	− 244.717	0.1645	Age (−)
Scrotal Redness ~ Age + Rank	**0.04591**	− 284.534	0.2483	Age (+)
Scrotal Luminance ~ Age + Rank	**0.009823**	− 209.346	0.3245	Age (−)
Log(T) ~ Age + Rank	0.3097	− 242.9525	0.06436	None

Significant *p*-values are in bold.

### Effect of androgen and color ornamentation on mating and reproductive output

Males with higher androgen concentrations showed higher rates of mounting and spent more time in consortships (Table 2), but ejaculation rates were not predicted by androgen concentrations. Mounting and consortship rates also varied in relation to color measures with males with redder scrotum having higher rates of mounting and males with darker scrotum spent more time consorting. Variation in ejaculation rates was not predicted by facial or genital redness or luminance. Group instability (i.e., being a top-ranking male in a group that was not well-established—coded as Rank 4) had a significant negative effect on consortship rates, meaning that males who were top-ranking in small, unestablished groups whose membership was not consistent had lower consortship rates.

**Table 2:** Models used to predict reproductive behavior variables in the rhesus macaques, *Macaca mulatta*, living on Cayo Santiago, Puerto Rico.

Model	*p* Value	AIC	R^2^	Significant effect
Consort ~ FaceRed + log(T) + Rank + Age	**0.03**	− 102.203	0.3513	logT(+); rank 4 (−)
Consort ~ FaceLum + log(T) + Rank + Age	**0.04079**	− 101.441	0.33	logT(+); rank 4 (−)
Consort ~ SRed + log(T) + Rank + Age	**0.01121**	− 104.031	0.4145	logT(+); rank 4(−)
Consort ~ SLum + log(T) + Rank + Age	**0.002**	− 108.219	0.4788	SLum (−); logT(+); rank 4 (−)
Ejac ~ FaceRed + log(T) + Rank + Age	0.7784	− 156.516	0.0964	None
Ejac ~ FaceLum + log(T) + Rank + Age	0.78008	− 156.494	0.09588	None
Ejac ~ SRed + log(T) + Rank + Age	0.6688	− 152.358	0.1234	None
Ejac ~ SLum + log(T) + Rank + Age	0.3971	− 154.879	0.1827	None
Mount ~ FaceRed + log(T) + Rank + Age	0.3591	− 120.925	0.1869	None
Mount ~ FaceLum + log(T) + Rank + Age	0.3019	− 121.629	0.2022	logT( +)
Mount ~ SRed + log(T) + Rank + Age	0.3119	− 122.866	0.07279	SRed( +); logT ( +)
Mount ~ SLum + log(T) + Rank + Age	0.1473	− 120.474	0.2646	logT( +)

Significant *p*-values are in bold.

Consort: Consortship rates; Ejac: Ejaculation rates; Mount: muting rates; FaceRed: Face redness; FaceLum: Face luminance; SRed: Scrotal redness; SLum: Scrotal luminance; log(T): logarithimization of testosterone concentrations. AIC: Akaike Information Criteria. Rank: Alpha (1), beta (2), and gamma (3) males in an established group (values 1–3) or males that were among the highest-ranking males of small, unestablished groups (i.e. value of 4).

The number of offspring sired by the focal males for the mating season in which they were observed was predicted by several measures (Table 3). Age was significant in all models, with older males siring fewer offspring. Males that sired more offspring had redder faces and both darker and redder scrotum. Finally, group instability was significant in all models, with high-ranking males from small, unestablished groups producing fewer offspring. The best fit model included the interaction between scrotum redness, testosterone concentrations, rank, and age (lowest AIC = 146.0; Nagelkerke’s R^2^ = 0.80).

**Table 3:** Models used to predict the number of offspring produced in the rhesus macaques, *Macaca mulatta*, living on Cayo Santiago, Puerto Rico.

Offspring produced in 2013	AIC	R^2^	*p*	Effect
FaceRed + log(T) + Rank + Age	**155.25**	**0.774654**		
FaceRed			** < 0.001**	+
log(T)			** < 0.001**	−
Age			** < 0.001**	−
Rank 2			0.652806	+
Rank 3			0.205734	−
Rank 4			** < 0.001**	−
FaceLum + log(T) + Rank + Age	**175.5902**	**0.605811**		
FaceLum			0.11319	−
log(T)			0.13593	−
Age			** < 0.001**	−
Rank 2			0.87418	−
Rank 3			0.37972	−
Rank 4			** < 0.001**	−
SRed + log(T) + Rank + Age	146.5989	0.808512		
SRed			** < 0.001**	** + **
log(T)			**0.0403**	−
Age			** < 0.001**	−
Rank 2			0.3969	** + **
Rank 3			0.9968	** + **
Rank 4			**0.0127**	−
SLum + log(T) + Rank + Age	151.203	0.781713		
SLum			** < 0.001**	−
log(T)			0.9347	+
Age			** < 0.001**	−
Rank 2			0.1913	+
Rank 3			0.6974	+
Rank 4			**0.0179**	−

Significant *p*-values are in bold.

FaceRed: Face redness; FaceLum: Face luminance; SRed: Scrotal redness; SLum: Scrotal luminance; log(T): logarithimization of testosterone concentrations; R^2^ refers to Nagelkerke’s R^2^.

## Discussion

Our study provides support for the hypothesis that facial and genital skin color in male rhesus macaques are sexually-selected signals associated with variation in reproductive success; it also supports the view that older male rhesus macaques experience reproductive senescence. Our results point to testosterone as a potential physiological mechanism underlying variation in these ornaments.

In our study, high-ranking males across all groups in the Cayo Santiago population were represented in the dataset, whereas previous research generally examined color variation in males within one group. Although previous research did not find an association of facial skin coloration and androgen concentrations^60^, our results indicate that these factors are associated in high-ranking males. These findings suggest that there could be an underlying factor associated with being high-ranking that interacts with testosterone in producing skin coloration, such that they are only correlated among high-ranking males, and that testosterone is not the only factor that affects skin coloration. Examples of potential underlying factors include variation in mating behaviors, genetic quality, or general health/body condition. Better condition may simultaneously enable higher testosterone, redder coloration, and higher rank to be achieved, which could explain the correlations observed. Such patterns have been observed in birds^80^ and mammals^81^, including primates specifically^22^.

We found that high-ranking males with redder scrotum had higher rates of mounting and males with darker scrotum had higher rates of consortship. In previous studies, rhesus macaque females paid more attention to and preferred to mate with males with dark red faces^33,53^; males exhibited darker faces on days that they were observed copulating than on days in which they did not copulate^54^. However, we did not find a significant relationship for facial coloration and mating behaviors, only between scrotal coloration and those behaviors. The lack of relationship between facial color and mating behaviors may be due to using monthly averages instead of daily or weekly measures. Higher testosterone concentrations were also associated with increased mounting by males and a higher proportion of days spent consorting. These results are consistent with previous findings that males with higher testosterone spend more time in consortships^58^. Although the directionality of the relationship between skin coloration, testosterone, and mating behavior is unclear, it is possible that if males with a particular skin coloration are preferred by females, the resulting increased opportunities for mating for these high-ranking males may trigger a feedback loop that results in higher circulating androgens. Alternatively, androgens may be a proximate factor mediating skin coloration in high-ranking male rhesus macaques, and the resulting trait for males with high circulating testosterone concentrations attracts females and leads to more mating opportunities.

We found that facial and genital skin coloration was associated with variation in the number of offspring sired by males. Males with dark red scrotal coloration and red faces sired more offspring. The strongest association in our study was between scrotal color and offspring sired. Males with dark red scrotums had more offspring than other males, consistent with the findings of Dubuc et al.^62^. Despite having dark facial and genital skin, the oldest males produced fewer offspring and had lower testosterone concentrations; this finding is consistent with a previous report of male reproductive senescence in this population, indicating that despite high mating effort, old males do not sire many offspring^61^. In vervet monkeys (*Chlorocebus* sp.), older males and males with larger canines had lighter scrotal color^82^. However, it is important to note that there is an interaction between color, rank, age, and testosterone, and understanding this relationship is complex under natural conditions, as was seen in wild birds^80^.

It is possible that skin coloration correlates with physical, morphometric measurements, but this relationship has not been assessed in rhesus macaques. More studies integrating experimental and naturalistic approaches are needed to provided evidence concerning the hormonal regulation of skin coloration in rhesus macaques. Given that our study was conducted on high-ranking males who generally have more mating opportunities than low-ranking males and we only collected data during the mating season, we may not have captured the full range of color variation that is seen across the entire population during all times of the year as well as the full range of its physiological, behavioral, and reproductive correlates. Future studies could further explore these relationships by assessing changes in high-ranking male coloration throughout the year and in relation to changes in behavior, physiology, and reproduction. Additionally, collecting daily fecal samples and color measurements during the peak-mating season would allow us to examine the timing of changes in androgens in relation to color changes to try to determine the directionality of that relationship. Moreover, with the availability of genetic paternity data, one could assess offspring survival in relation to the sire’s coloration at the time of conception.

In our sample of all high-ranking males, we did not find an association between male rank and any of the coloration measures for the three highest ranking males in each group. These findings are consistent with previous reports that found that male coloration and rank are not linked in rhesus macaques^33,60^. One previous study did find an association between rank and color in males, but only on days they copulated^54^. In some other primate species, a relationship between color and rank has been reported; for example, high-ranking male vervet monkeys exhibited a lighter scrotum color (*Chlorocebus pygerythrus*^25^) and alpha crested macaque males have the most colorful scrotum in their group (*Macaca nigra*^83^). Colorful ornamentations are hypothesized to function as badges of status or quality signals^21,30^ that reflect dominance status in some species^84,85^. However, in species where dominance is often acquired through age or tenure in a group, such as rhesus macaques, the dynamics between color and dominance status likely differ.

As previously reported for this rhesus macaque population^60^, we found a correlation between facial and genital skin redness and luminance. However, unlike Higham et al.^30^, we found a relationship between facial skin coloration and androgen concentrations, as well as that genital luminance (but not redness) co-varied with androgen concentrations in the highest-ranking males in the population. Since it was first noticed, red color in rhesus macaque males has been assumed to be androgen-dependent^18,36,39^ (but see Higham et al.^60^). Testosterone induces vasodilatation and increases blood flow^44,86^, resulting in more intense scrotal redness^44,87^. In a study in which male rhesus macaques were treated with exogenous hormones, scrotal redness increased after androgen administration, but there was no effect on facial redness^44^. In some immunocastrated males (e.g., lambs), the resulting reduction in circulating androgens was associated with darker and redder scrotal relative to control animals that did not undergo immunocastration^88^. Such differences in directionality of the relationship between androgens and redness may reflect species differences in female preferences, but the links between androgen concentrations and color suggest that this trait is under hormonal control. More research is needed to precisely understand the nature of the cause-effect relationships.

Androgens are not the only hormone to mediate color. In a number of different primate species, female sex skin color is known to be dependent on hormones, particularly estrogens^89–92^. The same is presumably true also for female facial coloration, which in some species such as rhesus macaques, becomes redder around the time of ovulation^72,93^. Future research on color ornamentation in male rhesus macaques should incorporate measures of estrogens, as well.

Facial color in male rhesus macaques has been hypothesized to be linked to female mate choice^94^. In rhesus macaques, males with dark red faces receive as much attention from females (as well as from other adult males^53^) as high-ranking males do^33,54^. Moreover, although rhesus macaques exhibit linear dominance hierarchies related to age classes for males^45^, top-ranking males do not necessarily have redder faces or genitals^30,33,52^. Our study confirmed that higher rates of mating behaviors and offspring sired were indeed independent of top rank position, but they did correlate with coloration. Although we did not directly measure female mate choice, our study combined with these previous studies of the same population^30,33,52–54^ support the hypothesis that male coloration is a sexually selected trait that is linked to female mate choice.

Alternatively or in addition to female mate choice, skin coloration in rhesus macaques could be a sexually-selected signal that influences male-male competition. When competition is intense, trait expression and dominance can be correlated (e.g., orang-utan cheeks^95^; vervet scrotal color^28^). Such displays are thought to prevent escalation to physical aggression between males^96^. If these traits function to signal individual quality, intrasexual competition may be enhanced by mate choice as those “winner” traits may also be preferred by females^3,7^, as is the case in mandrills^34^ and could also be applicable to our study. Male coloration may be an honest signal of body condition and health that females can use to select mates^3,4,20^. Our study, in combination with previous research, provides support for the hypothesis that male facial color is linked to female mate choice in rhesus macaques, and we provide evidence that genital ornamentation—particularly luminance—is also a key element in this mix and that androgens are associated with variation in these color variables in high-ranking male rhesus macaques.

Given previous evidence of declining reproductive function with older age in a variety of animal species^97^, changes in sexually-selected traits in males may be a widespread phenomenon. These age-related changes in color ornamentation could result from reduced hormone concentrations, fewer mating behaviors, or both. In our study, however, relationships are complex, with oldest males having dark faces and genitals and engaging in mating behaviors despite their lower reproductive output. The oldest males (age 18 and above) were sometimes but not always the alpha of their groups. These oldest males had variable testosterone concentrations, consorted frequently, but sired a maximum of one offspring each in 2013. Understanding the relationship between reproductive senescence and sexually-selected traits is an important area of research in evolutionary biology, as it can shed light on the factors that shape mating behavior and reproductive success in male vertebrates.

## Data Availability

The datasets generated and/or analyzed during the current study are available in a repository, https://1drv.ms/x/s!AhcbdRJNpLfrhttjPcEN7M6zFaKdIg?e=hWPbAO.
